
JOURNAL INFORMATION
==============================
NLM Title Abbreviation: ScientificWorldJournal
Journal ID: ScientificWorldJournal
NLM Journal ID: 101131163
Journal ID: TSWJ

The Scientific World Journal

ISSN: 2356-6140
EISSN: 1537-744X
Publisher: Wiley

ARTICLE INFORMATION
==============================
PMCID: PMC6084138
PMID: 30147618
DOI: 10.1100/tsw.2001.238
Article ID: 486743
Article version: 1
Subjects: Short Report

Aged Yeast Mother Cells Show Markers of Apoptosis

Laun Peter *
Pichova Alena
Madeo Frank
Ellinger Adolf
Kohlwein Sepp D.
Froumlhlich Kai-Uwe
Dawes Ian
Breitenbach Michael
Institut für Genetik und Allgemeine Biologie, Hellbrunnerstraße 34a, 5020 Salzburg, Austria
*Peter Laun: peter.laun@sbg.ac.at
Publication date: 2001
Electronic publication date: 2001 Dec 12
Volume: 1
First page: 141
Last page: 141
Copyright: Copyright © 2001 Peter Laun et al.
Copyright year: 2001
License: This is an open access article distributed under the Creative Commons Attribution License, which permits unrestricted use, distribution, and reproduction in any medium, provided the original work is properly cited.
License URL: https://creativecommons.org/licenses/by/3.0/

Funding: Austrian Science Fund P14574-MOB

==============================
Miami Nature Biotechnology Short Reports

TheScientificWorld (2001) 1 (S3), 141SR

ISSN 1532-2246; DOI 10.1100/tsw.2001.238

### AGED YEAST MOTHER CELLS SHOW MARKERS OF APOPTOSIS

Peter Laun*, Alena Pichova, Frank Madeo, Adolf Ellinger, Sepp D. Kohlwein, Kai-Uwe Fröhlich, Ian Dawes, and Michael Breitenbach

Institut für Genetik und Allgemeine Biologie, Hellbrunnerstraße 34a, 5020 Salzburg, Austria
* Peter.Laun@sbg.ac.at

**INTRODUCTION.** Recently, others and we have shown that genetic and environmental changes that increase the load of yeast cells with ROS (reactive oxygen species) lead to a shortening of the life span of yeast mother cells (1). Deletions of yeast genes coding for the superoxide dismutases or the yeast catalases, as well as changes in atmospheric oxygen concentration and the presence of the physiological antioxidant, glutathione, all had the expected distinct effects on lifespan and pointed to a role of oxygen in the yeast aging process. We have now undertaken a cytological and biochemical characterization of old yeast mother cells prepared by the elutriation method (Molecular Microbiology, in press 2001).

**METHODS.** We have developed a new method for bulk isolation of old and young yeast cells by elutriation. The method is based on cell size only but yields remarkably pure old and young cell fractions. To characterize these cells, we used standard methods of fluorescence microscopy staining for ROS with dihydrorhodamine123 (DHR). The TUNEL methods as well as staining with annexinV were used to detect markers of apoptosis. Fluorescent staining with Calcofluor White was used to reveal bud scars. Life spans were determined by micromanipulation as described (2).

**RESULTS.** The fraction containing large cells had a median lifespan of about 3.5 generations. About 10-20% of these cells were in their last cell cycle. Cells with more than 20 bud scars could be seen. The cytoskeleton was frequently abnormal pointing to loss of cell polarity. The fraction containing small cells in the same strain displayed a median lifespan of about 25 generations identical to the standard lifespan of the strain. The small cells showed just one birth scar and were normal in nuclear morphology.

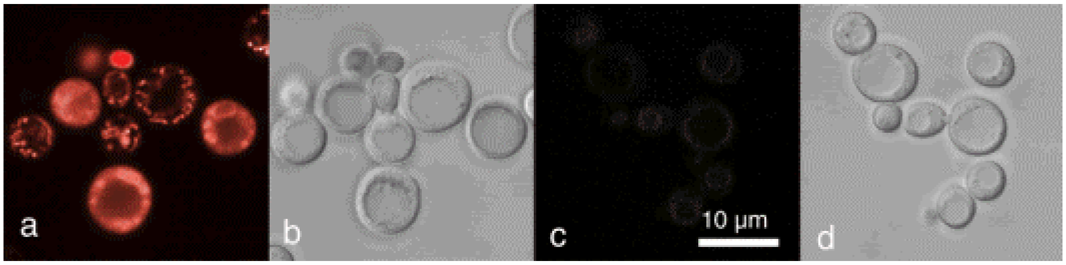

**Fig. 1.** DHR staining of old (a,b) and young (c,d) cells with fluorescence (a,c) and light microscopy (b,d).

Although the isolated old mother cells are wild type and have not undergone external oxygen stresses, these cells do contain a large amount of oxidizing species (ROS) in their mitochondria (Fig. 1a) while control cells (young cells prepared by the same method) do not (Fig. 1c). Moreover, 10-20% of the old cells undergo apoptosis as shown by the TUNEL and annexin V tests (3). Again, the control cells did not show apoptotic markers.

**DISCUSSION.** The present results further support the notion that oxygen toxicity may be a common feature of aging processes in a wide variety of eukaryotic cells. In addition, our finding of a primitive form of apoptosis in aged wild type yeast cells now enables us to further investigate the relationship between aging, oxygen stress and apoptosis in eukaryotes by yeast genetic methods. Recently it was shown that also primary human cells in culture undergo apoptosis during senescence (Jansen-Dürr, pers. comm.).

**ACKNOWLEDGEMENT.** This work has been supported by grant No. P14574-MOB (FWF Austria; to MB), an ARC IREX grant to IWD and MB and grant GA CR(CZ) GA204/97/0541 to AP.

**REFERENCES.**

1. Nestelbacher, R., Laun, P., Vondrakova, D., Pichova, A., Schuller, C., and Breitenbach, M. (2000) Exp. Gerontol. 35, 63-70
2. Pichova, A., Vondrakova, D., and Breitenbach, M. (1997) Can. J. Microbiol. 43, 774-781
3. Madeo, F., Frohlich, E., Ligr, M., Grey, M., Sigrist, S.J., Wolf, D.H., and Frohlich, K.U. (1999) J. Cell Biol. 145, 757-767
